# Technology Advancements in Blood Coagulation Measurements for Point-of-Care Diagnostic Testing

**DOI:** 10.3389/fbioe.2019.00395

**Published:** 2019-12-11

**Authors:** Mohammad Mohammadi Aria, Ahmet Erten, Ozlem Yalcin

**Affiliations:** ^1^Graduate School of Biomedical Sciences and Engineering, Koc University, Sariyer, Turkey; ^2^Department of Electronics and Communication Engineering, Istanbul Technical University, Istanbul, Turkey; ^3^Department of Physiology, Koc University School of Medicine, Koc University, Sariyer, Turkey

**Keywords:** blood coagulation, POC devices, electrochemical sensing, MEMS, fluorescent microscopy, microfluidics, nanomaterials, photoacoustic detection

## Abstract

In recent years, blood coagulation monitoring has become crucial to diagnosing causes of hemorrhages, developing anticoagulant drugs, assessing bleeding risk in extensive surgery procedures and dialysis, and investigating the efficacy of hemostatic therapies. In this regard, advanced technologies such as microfluidics, fluorescent microscopy, electrochemical sensing, photoacoustic detection, and micro/nano electromechanical systems (MEMS/NEMS) have been employed to develop highly accurate, robust, and cost-effective point of care (POC) devices. These devices measure electrochemical, optical, and mechanical parameters of clotting blood. Which can be correlated to light transmission/scattering, electrical impedance, and viscoelastic properties. In this regard, this paper discusses the working principles of blood coagulation monitoring, physical and sensing parameters in different technologies. In addition, we discussed the recent progress in developing nanomaterials for blood coagulation detection and treatments which opens up new area of controlling and monitoring of coagulation at the same time in the future. Moreover, commercial products, future trends/challenges in blood coagulation monitoring including novel anticoagulant therapies, multiplexed sensing platforms, and the application of artificial intelligence in diagnosis and monitoring have been included.

## Introduction

The circulating blood inside our bodies has many functions including transporting O_2_, CO_2_, and delivering nutrients to the cells. This circulating blood is also a significant source of information on *in vivo* coagulation parameters, hypercoagulability, and alterations in fibrinolysis. Blood coagulation involving a blood fluid to become a solid clot is critical to stop bleeding when an injury occurs inside or outside of the body. However, abnormalities in blood coagulation such as hypercoagulability can cause excessive blood clots and vein blockage, leading to stroke (Kahn, 2003; Levine, 2005). Cancer (Falanga et al., 2013), infectious diseases such as HIV (Lijfering et al., 2008) and hepatitis, trauma (Rugeri et al., 2007), diabetes, and retinal vein occlusion (Kuhli-Hattenbach et al., 2010; Goren Sahin et al., 2018) etc. affect the coagulation stages and create serious complications, as well. For example, the tumor cells can cause thromboembolic complications due to activation of blood coagulation by producing procoagulants such as tissue factor (the primary activator of blood coagulation), releasing soluble factors such as thrombin that induce platelet activation, aggregation, expressing proteins regulating the fibrinolytic system and causing impairment in plasma fibrinolytic activity (Marinho and Takagaki, 2008). Therefore, accurate measurement and understanding of hemostasis including blood coagulation and fibrinolysis is highly demanded to study defects from sensing parameters in different disease models.

Blood coagulation and fibrinolysis are complex processes in which platelets, fibrins, enzymes, and a series of complex chemical reactions play a role. Since red blood cells (RBCs) also have significant functions in blood clotting and its disorders (Demiroglu, 1997; Yalcin et al., 2016; Litvinov and Weisel, 2017), the RBC rheology in different disease conditions has been widely studied (Ertan et al., 2017; Ugurel et al., 2017; Yalcin et al., 2017). For example, red blood deformability is controlled by calcium (Yalcin et al., 2016) and it directly affects the blood viscosity and clotting. In addition, the force provided by clotting is high enough for the 10 pN upper force limit needed for RBC aggregation triggered by Ca^+^ (Bernhardt et al., 2019). The coagulation process begins with vascular and platelet phase (primary hemostasis), continues with activation of the coagulation cascade (secondary hemostasis) and ends when the clot dissolves through the fibrinolytic system (fibrinolysis). In the primary hemostasis, after the endothelium cells (vascular inner surface) are injured, procoagulant subendothelial matrix (such as laminin, collagen, and fibronectin) will be exposed to initiate primary phase. Then, the platelets come in contact with the collagen that occurs on the surface of the vessel, and release of adhesive proteins such as von Willebrand factor (vWf) from the endothelial cells leads to adhesion of platelets to the exposed subendothelial matrix. Subsequently, the adhesion of platelets causes platelet activation which will be amplified by platelet activator thrombin generated by coagulation cascade. The secondary hemostasis includes the stages of consolidation of the platelet plug and formation of fibrin clot. Coagulation proteins are the main components of the coagulation system, leading to a complex sequence of reactions and conversion of soluble fibrinogen to insoluble fibrin strands.

**Figure 1:**
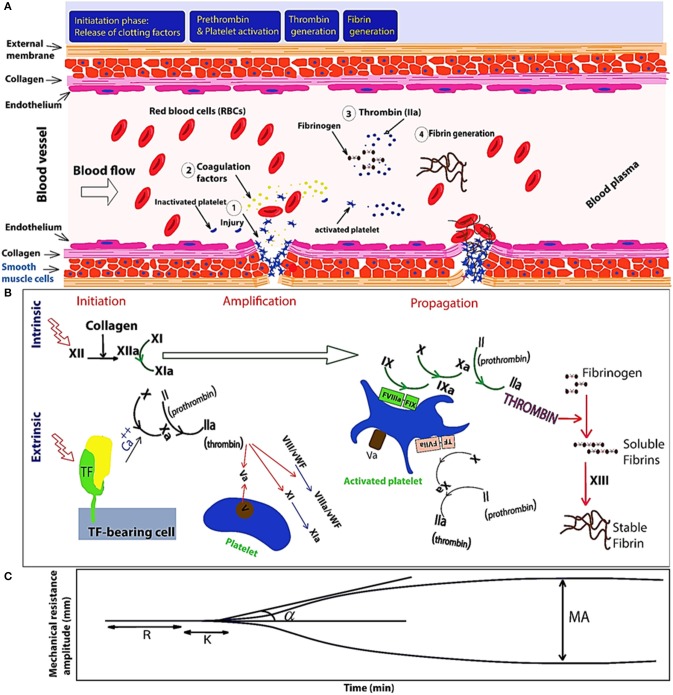
Schematic of the primary and secondary phases of hemostasis. **(A)** Initiation of hemostasis by an injury to a blood vessel. **(B)** Illustrates three phases of initiation, amplification (platelet activation happens during the initiation phase, and is probably mediated through multiple platelet signaling pathways and thrombin), and propagation triggered by extrinsic and intrinsic pathways. Extrinsic pathway is triggered by tissue factor (TF)-activated coagulation factor VII (fVIIa) and consequently TF-fVIIa complex triggers Ca^++^ (calcium) ion-dependent enzymatic reactions in response to injury of blood vessels composed of endothelial cells and the vessel wall. Intrinsic pathway also is initiated by contact with collagen and XII will be activated as XIIa (a = activated). When XIIa is present, factor XI will be activated. Then, XIa will activate factor IX. Also, factor Xia together with factor VIII will convert factor X into Xa. Finally, Xa will activate the Prothrombin-activator; triggering the Common Coagulation Pathway. Von Willebrand factors (vWFs) bind factor VIII, which is a key clotting protein, and it helps in forming a platelet plug during the clotting process. **(C)** TEG signal at the bottom shows the changes in the viscoelastic properties vs. time. Reaction time (R) represents delay time from test initiation until beginning of fibrin formation, measured as an increase in amplitude of 2 mm. The clotting time (K) is the time to clot formation, measured from the end of R until an amplitude of 20 mm is reached. The angle (a) represents the kinetic of fibrin accumulation and bonding. Maximum amplitude (MA) represents clot strength.

Figure 1 illustrates blood coagulation process including enzymatic cascades and evolution of the related TEG signal, in different stages of coagulation. As shown in the Figures 1A,B, the enzymatic reaction is initiated by the intrinsic or extrinsic activators and finally leads to common pathway. At the beginning of the process, the complex form of the tissue factor and factor VII activates factor IX, which demonstrates that the intrinsic and extrinsic coagulation pathways are interconnected. Second, the remained process includes three consecutive phases: the initiation phase, an amplification phase, and the propagation phase. In the two last phases, both platelets and thrombin are actively contributing (Thiruvenkatarajan et al., 2014). In the intrinsic pathway, negatively charged surfaces stimulate blood clotting with factors XII, XI, IX, and VIII. In extrinsic pathway, release of tissue factor initiates blood clotting with factor VII. And finally, the common pathway is triggered by factor X and it results in fibrin generation with factor V, prothrombin, and fibrinogen. Produced soluble fibrins are converted to stable fibers through factor XIII. Figure 1C shows the mechanical resistance of clotting blood during coagulation process. To study the disorders in the coagulation cascade, time response of the mechanical changes of clotting blood are compared with the ranges recorded from healthy donor and patient bloods in databases. Thromboelastogram (TEG) and rotational thromboelastometry (ROTEM) have been widely used as rheometers adapted for clinical application of blood coagulation monitoring (Ganter and Hofer, 2008). To monitor blood coagulation, observing mechanical properties of the clotting blood is the most powerful method. Mechanical changes in the clotting blood (viscosity and shear resistance vs. time) are correlated to the different parameters in the coagulation process such as clotting times and clot formation. In fact, the blood clot formation is monitored when it is resisting an applied shear stress. In addition, the blood viscosity or shear resistance is affected by the shear rate and it should be monitored in physiological range of shear rates similar to those inside the body to prevent disturbance of the coagulation process by platelet aggregation (Heemskerk et al., 2002; Ranucci et al., 2014). Coagulation can be initiated by an intrinsic stimulator reagent such as ellagic acid/phospholipid (Bock et al., 1981), by the extrinsic stimulator reagent such as tissue factor (Camerer et al., 1996), or fibrinolysis can be stimulated by the platelet inhibitors such as cytochalasin D in combination with extrinsic stimulator (Nielsen et al., 2000).

In previous reviews (Ganter and Hofer, 2008; Harris et al., 2013a) various point of care (POC) devices for blood coagulation tests have been compared and their working mechanism of detection briefly discussed, while in this paper blood coagulation monitoring techniques covering recent emerged technologies (till 2019) from engineering perspective have been presented. Recent technical advances and developments has been covered by emphasis on the mechanisms of the blood coagulation measurements with different technologies in order to provide an insight to researchers on advantages of certain techniques in detecting disorders and future trends for clinical monitoring and treatments. Moreover, the role of nanomaterials for both detection and treatment has been discussed, and finally available commercial devices, future trends about monitoring of novel anticoagulant drugs, artificial intelligence in diagnosis, and monitoring for higher accuracy of measurements have been described. Therefore, both areas of academic and industrial sections have been covered which enables to fill the current gaps in the blood coagulation monitoring and possible developments for continuous hemostasis monitoring in the future.

## Point-of-Care Testing for Coagulation Monitoring

Point-of-care (POC) tests for coagulation monitoring with the help of various activators, play an important role in detecting bleeding disorders and monitoring of anticoagulant drugs. Prothrombin time (PT), activated partial thromboplastin time (aPTT), thrombin time (TT), Prothrombinase induced clotting time (PiCT) are the most useful tests to investigate patients with coagulopathies and drug monitoring (Kamal et al., 2007; Hussain, 2015). These tests are used to monitor safety and effectiveness of anticoagulants, and sensitive to the lack of many coagulation factors. PT test is used to investigate clotting blood from extrinsic and common pathways in which factors I (Fibrinogen), II (Prothrombin), V, VII and X contribute. APTT test is measuring clotting from intrinsic and common pathways. In summary, PT is sensitive to factors II, V, VII, and X deficiencies, whereas aPTT is sensitive to deficiencies of prekallikrein, high molecular weight kininogen (HMWK), factors XII, XI, IX, and VIII. In addition, PT and aPTT are also sensitive to the presence of circulating anticoagulants such as lupus (Rosner et al., 1987). To standardize PT values, International Normalized Ratio (INR) is used which is mathematically driven from PT to include reagent sensitivity and varying instrument to keep the patients within narrow therapeutic ranges. INR = (patient PT/patient MNPT) ^ISI^ represents the formula to calculate INR, where the MNPT is the mean normal PT, and the ISI is an international sensitivity index (a factor to adjust instrument and reagent sensitivity differences). The therapeutic or conventional INR ranges are between 2.0 to 3.0. INR levels above 4.9 are examined as critical values and increasing the risk of bleeding. As an alternative, a narrow INR range lower than 2.0 (1.5 to 1.9) is used to reduce bleeding risks and so improve the safety profile of anticoagulation (Secretariat, 2009; Ong et al., 2016; Guimarães et al., 2019).

The biggest appeal of POC devices is short measurement durations (25–45 min) compared to conventional coagulation tests (40–60 min). POC INR devices can also be used in pre-hospital emergency services to provide fast and reliable results for patients suspected of coagulation factor deficiencies (Niederdöckl et al., 2016). To perform the aforementioned coagulation tests, different technologies such as electrical impedance spectroscopy, micro/nanoelectromechanical (MEMS/NEMS) resonator based rheometers, optical and photoacoustic measurements, and microfluidic viscometers have been employed. In this regard, understanding working principle and mechanism of these techniques facilitates researchers in identifying proper setup for laboratory or clinical applications, current gaps, and designing future devices. Among these technologies, photoacoustic/ultrasound measurements (Huang et al., 2005a; Huang C.-C. et al., 2011; Scola et al., 2011; Das and Pramanik, 2019) and microfluidic platforms have been used for *in/ex vivo* tests which are very promising for translational medicine when actual monitoring of the drug/anticoagulant effects or detection of circulating blood clots (CBCs) is desired (Karpiouk et al., 2008; Jain et al., 2016a).

Table 1 lists different technologies including optical, electro-mechanical, photoacoustic, and electrical impedance spectroscopy for blood coagulation measurement, physical parameters, and their use in coagulation tests. Optical blood coagulation measurement techniques include laser speckle rheology (LSR), optical coherence elastography (OCE), fluorescent imaging, and surface plasmon resonance (SPR). While viscoelastic properties and shear modulus are correlated to the blood coagulation parameters in LSR (Tripathi et al., 2014) and OCE (Xu et al., 2019), the fluorescent intensity level and refractive index are measured for studying clotting parameters in fluorescent imaging (Dudek et al., 2010) and SPR (Hansson et al., 2007), respectively. The second category of the blood clotting measurement devices involve electromechanical technologies covering all types of MEMS/NEMS resonators, microfluidic viscometers, and ultrasound detection systems. The third category is photoacoustic detection based on applying a laser beam and detecting the emitted acoustic signal (thermoelastic wave). In this technology, a photoacoustic (PA) signal enables monitoring of the adherent thrombi and emboli in deep tissue (Juratli et al., 2016). As optoacoustic signals provide high accuracy measurements of physiologic variables at depths greater than the optical diffusion limit, it is a powerful tool for recording the physical changes of tissue by monitoring the effective absorption coefficient (**μa**), effective attenuation coefficient (**μeff**), and the thermoacoustic efficiency (**Γ**) parameters (Wang, 2009). The fourth and the most widely used technology for measuring clotting time in POC tests is electrochemical impedance spectroscopy technique since it is low cost and simple to implement. This technology employs integrated circuits such as electronic amplifiers and digital synthesizers to measure impedance change of clotting blood with high accuracy. Second category of this technology is electrochemical aptamer biosensors for thrombin measurement (Mir et al., 2006).

**Table 1:** Summary of the blood coagulation measurement techniques and physical parameters in each technique in research methodologies.

**Technique**		**Physical parameter**	**Sensing parameter**	**Applications**	**References**
Optical	LSR	G: viscoelastic modulus τ: time constant of laser speckle intensity fluctuations	Significant correlation between τ and |G*| (*r* = 0.79, *p* < 0.0001)	PT and APTT	Tripathi et al., 2014
	OCE,OCT	μ: shear modulus	Viscoelastic peroperties	Clot shear modulus	Xu et al., 2016
	Fluorescent imaging	Fluorescent intensity	Heparin and tinzaparin from 0 to 0.8 U/ml	APTT, PT Thrombodynamics analysis (TD)	Dudek et al., 2010; Yun et al., 2019
	SPR	n_s_: refractive index	7 to 8 ng/mm^2^ cell adhesion detection	PT	Hansson et al., 1999, 2007
Electro-mechanical	Acoustic wave devices (QCM and SAW)	1. Frequency shift as a result of mass change (μg) in liquid Δ*_*m*_* 2. Dissipation factor in liquid Δ*_*D*_*	Factor VIII: 0.0185 mg/L Fibrinogen: 1 g/Lf	PT, APTT, and INR	Rodahl et al., 1995 Yao et al., 2013 Hussain, 2015
	Microfluidic viscometers	Pressure (Pa) and mechanical displacements	~0.4–12 p.s.i. Heparin detection: 0.25 IU/mL	PT, APTT, ACT, and INR	Jain et al., 2016a; Hegener et al., 2017; Yeom et al., 2017; Judith et al., 2018 Kang et al., 2019
	MEMS (Magneto elastic cantilevers)	Viscosity	Blood viscosity: 0.08 cp	PT, APTT, and INR	Cakmak et al., 2015
	Ultrasound transducers	Sound velocity (m/s) Displacement (μm)	Sensitivity of 3 cm/s	Viscoelastic test–derived coagulation parameters	Voleišis et al., 2002; Huang C.-C. et al., 2011; McLoughlin et al., 2016
Photoacoustic detection		Photoacoustic signal (PA intensity) μ_a_, μ_eff_, Γ	0.28 IU/mL of heparin detection	APTT	Wang et al., 2016
Electrochemical (Amperometric, impedance analysis)		Conductance, electrical current (amplitude/phase) [Ohm]	0.33 fM (thrombin detection)	PT, APTT, and INR	Lei et al., 2013; Liu et al., 2018

### POC Tests Based on Optical Measurements

Viscoelastic properties of blood can be monitored by studying its optical properties either by through light scattering or light transmission. In laser speckle rheometers, light scattering due to the Brownian motion in the blood gives direct information about viscoelastic properties or coagulation of the blood (Weitz and Pine, 1993). In order to measure this, the time-varying speckle intensity of fluctuations is measured by finding autocorrelation curve from a series of image frames while the clotting blood is illuminated with a laser (Hajjarian and Nadkarni, 2012; Tripathi et al., 2014). LSR method also suggests a rapid assessment of anticoagulation status in real-time.

In OCE system, map of tissue stiffness and spatially distributed mechanical displacements on the micrometer to millimeter scale is achieved by the shear modulus measurement of a propagated shear wave in the blood sample (Kennedy et al., 2014; Xu et al., 2019). In fact, a shear wave source via a remote ultrasonic transducer provides us with a mechanical shear wave in the blood sample. Consequently, blood coagulation parameters including clotting times and stiffness are related to the velocity of the mechanical wave and the shear modulus of clotting blood (Xu et al., 2016).

High sensitivity of refractive index measurement thanks to SPR in plasmonic metal particles facilitates the detection of cell adhesion at the interface as low as 7 ng mass changes (Hansson et al., 2007). The refractive index in the metallic particles is changing around the plasmon resonance and it depends on both wavelength and angle of incidence. To measure the refractive index at the plasmonic interface, a light emitting diode (LED) or laser is used to illuminate a planar light wave while a photodiode detects the reflected light intensity. Therefore, coating of the plasmonic nanoparticle based interfaces with different agents enables the detection of different elements in the blood coagulation process such as platelets, fibrins, RBCs in the blood. Plasmon resonance in metal particles have been used widely for biosensing applications and it is a very promising technology for multiplexed sensing (detection of different antigen, chemicals, and blood coagulation on the same platform). The detection of blood coagulation and platelet adhesion based on a plasmon resonance was first reported by Hansson et al. (2007). In another study, Hayashi et al. employed plasmonic detection to measure the flow rate change of clotting blood. As the blood coagulates, the flow velocity decreases and the refractive index of the metallic interface also changes as the clotting blood travels at the interface slowly (Hayashi et al., 2012).

One of the big advantages of the fluorescent imaging is that it enables visualization of the dynamics of blood clot, analysis of blood coagulation with different fluorescent probes, and multiplexed sensing in the blood coagulation process. Fluorescent labeling techniques have drastically improved thrombosis monitoring by using fluorophores to track clot molecules and cells for quantitative measurement of multiple targets simultaneously. Dudek et al. described clotting time assay by fluorescent probing to detect onset of fibrin clot formation with high accuracy (Dudek et al., 2010). Since fluorescently labeled fibrinogen makes the study of platelet activation and fibrinogen binding possible, it is widely used for *in vivo* localization of clot formation (Heilmann et al., 1994). Falati et al. employed multiple fluorescent probes for real-time *in vivo* monitoring of platelet, fibrins, and tissue factor during arterial thrombus formation (Falati et al., 2002). Using near-infrared fluorescent factor XIII (FX13) probe, Tung et al. developed new types of imaging sensors promising for *in vivo* coagulation measurements (Tung et al., 2003). FX13 is a critical component at the final stage of blood coagulation, which causes significant stiffness and needed resistance against thrombolytic enzymes by adding plasmin inhibitors into the clot. Yun et al. explained fluorescent thrombin detection method based on DNA motor and proximity ligation assay (PLA) (Yun et al., 2019). In this method fluorophore labeled DNA is modified on gold nanoparticles (AuNPs) which quench the florescent signal, while Mg^2+^-dependent DNAzyme is produced by PLA between target thrombin and two aptamers. DNAzyme cleave circularly the fluorophore labeled DNA leading to release of fluorescent fragment from AuNPs' surface. Linear range in this method is 10 pM−10 nM with a detection limit of 4 pM (Yun et al., 2019). Liu et al. used carboxyl-functionalized semiconducting polymer dots (Pdots) as fluorescent donors along with a black hole quenching dye (BHQ-labeled thrombin aptamers) for sensing of thrombin based on fluorescent energy transfer mechanism (Liu et al., 2018). In this study, the correlated fluorescence intensity to thrombin concentration is in a linear range of 0–50 nM (*R*^2^ = 0.990) and a detection limit of 0.33 nM.

As microfluidic devices enable measurements in similar biological conditions and dynamic shear environment within the body, they are promising for quantitative analysis and simulating of the hematologic and vascular process such as thrombosis. In some studies, microfluidic devices embedded with a fluorogenic substrate are used for thrombin time detection and anticoagulant therapy assay. Leanne F. Harris used a microfluidic device integrated with a fluorogenic FXa substrate capable of measuring unfractionated heparin and tinzaparin from 0 to 0.8 U/ml (Harris et al., 2013b). Yu et al. demonstrated real time thrombin measurement based on droplet microfluidic technology by using fluorogenic substrates (Yu et al., 2014). Jain et al. designed microfluidic devices embedded with fixed endothelium to study platelet function and thrombus formation in physiological shear rate similar to a living arterial vessel. While intact fixed normal endothelium cells prevent clotting, the prestimulated cells with TNF-α promotes effectively thrombosis *in vitro* (Jain et al., 2016b). Flowing agonists responsible for the platelet activation and aggregation was studied by using a membrane-based microfluidic device which enables controlling the agonists' flux in the flowing blood (Neeves and Diamond, 2008). Jevgenia et al. employed a multi-bypass microfluidic ladder network by using light and fluorescent microscopy to study thrombus formation properties for which temporal distribution of platelet aggregation and fibrin formation was analyzed for different blood flow, shear gradients, and platelet/RBC distribution (Zilberman-Rudenko et al., 2017).

### POC Tests Based on Electromechanical Measurements

MEMS and NEMS devices have emerged over the past decade in POC platforms for sensing of different chemicals such as blood glucose (Kim et al., 2001; Huang et al., 2009), detection of Hepatitis viruses (Timurdogan et al., 2011), and blood coagulation tests (Cakmak et al., 2015). Capability of this technology in biosensing enables future multi sensing applications in blood. Although, in case of determination of the blood coagulation parameters, mechanical properties are measured through MEMS resonators in contact with the clotting blood allowing the extraction of changes of mechanical properties directly from the resonance signals. Damping factor changes as the viscoelastic parameters of the blood is altered under coagulation process. By decorating the resonators with specific antennas (antibodies), the possibility of measuring different protein adsorption and viscosity of the blood fluid separately is achievable. Nano-oscillation of MEMS resonators was used for the coagulation monitoring (aPTT and anticoagulant tests); which showed a very low detection limit of the 0.08 cP for blood viscosity measurement (Padovani et al., 2017). In another study, Cakmak et al. proposed MEMS-based sensor arrays enabling multiple clot-time tests (PT and aPTT) demonstrating a low cost cartridge based platform with a remote readout by applying magnetic field to actuate the resonators and detecting the displacements (resonance) by a laser (Cakmak et al., 2015).

One of the techniques to measure the viscoelastic properties of a fluid is detecting resonance parameters of electromechanical resonators such as acoustic wave devices while their surface is in contact with the fluid. For example, quartz crystal microbalance (QCM) is one of the widely known acoustic wave devices which has been widely used in bio/chemical sensors for ethanol gas sensing (Aria et al., 2016), glucose sensing (Saraoglu and Kocan, 2009), and for blood coagulation monitoring (Guhr et al., 2005; Hussain, 2016a). A QCM device consists of a piezoelectric material, which is sandwiched between two electrical contacts, is ultrasensitive to mass (μg scale in liquid environment) and viscosity-density ($8.5\times 10^{-4}\sqrt{g}/cm^{3}$) at its surface. Upon applying an electrical signal at its resonance frequency, it starts to generate electromechanical resonance. The resonance frequency and damping factors are affected by changing mechanical parameters of the electrode at the surface and other connected layers on top of which can be solid or a fluid like blood (Johannsmann, 2008). The viscoelastic changes in blood alter the resonance parameters such as frequency and dissipation (damping factor), and this analysis is called quartz crystal with dissipation (QCM-D). Moreover, since the surface of the electrode can be modified chemically with different types of ligands and proteins, the application of QCM-D to study hemostasis parameters such as platelet aggregation (Sinn et al., 2010), quick time prothrombin time (Cheng et al., 1998; Muller et al., 2009; Hussain, 2016b), prothrombinase induced clotting time (PiCT) (Hussain, 2015), tissue factor effect on the blood coagulation (Lakshmanan et al., 2016), determination of fibrinogen (1–6 g/L) and heparin concentration (Lakshmanan et al., 2013), detection of Factor VIII (Yao et al., 2013), and real time measurements of blood coagulation density and immune complement activation on artificial surfaces have been previously reported (Andersson et al., 2005). During the blood coagulation process, when the fibrins are generated from fibrinogen decomposition due to thrombin, blood viscosity increases and the frequency shifts both because of dissipation and increased mass absorption at the surface of the QCM enabling real time monitoring of blood coagulation. Another type of piezoelectric transducers is surface acoustic wave (SAW) devices. SAW devices are highly sensitive to surface mass changes (as a generated acoustic wave travels on the surface of a piezoelectric substrate), and their surface can be modified with aptamers for monitoring of a complex formation in blood coagulation process. The detection limit of this device for reversible protein bonding to an aptamer on the device surface has been reported as 75 pg/cm^2^ (Schlensog et al., 2004). Gronewold et al. employed array of coated SAW transducers with RNA and DNA aptamers for specific binding of human thrombin and thrombin inhibitors for their quantification in the presence of a variety of components in blood coagulation cascade (Gronewold et al., 2005).

**Figure 2:**
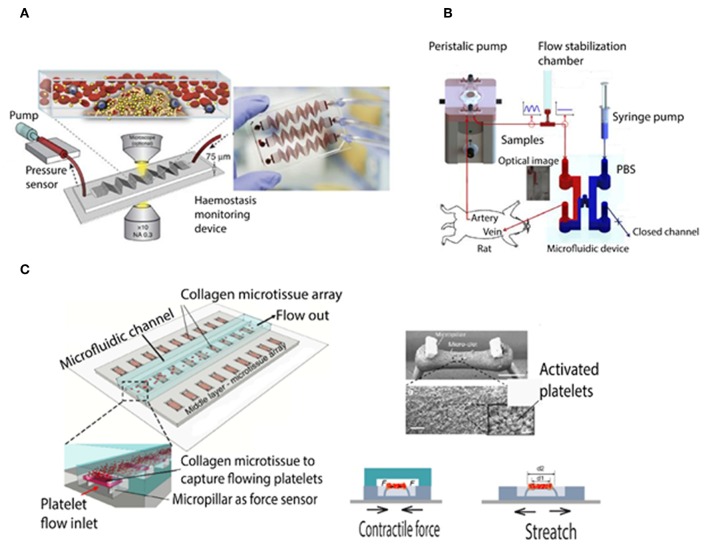
Schematic of microfluidic viscometer based blood coagulation monitoring devices. **(A)** Shear gradient-activated microfluidic device consisting of a syringe pump generates blood flow and an inline pressure sensor that is connected to the device via tubing measures the pressure to determine micro-clotting time. Fluorescence microscopy of fibrinogen and platelets also enables monitoring of thrombus formation at the same time (Jain et al., 2016a). **(B)** Schematic of the blood coagulation measurement system including a peristaltic pump, a microfluidic device, and a flow stabilizer. As the pressure and blood viscosity is correlated to the interfacial changes, an interfacial line between the PBS solution and blood sample is induced by closing the outlet of the PBS flow (Yeom et al., 2016). **(C)** Schematic of the micropillar arrays and microclot formation under platelet flow. Right-top shows the SEM image of two pillars and the microclot which formed between them (with 200 μm scale bar). Right-bottom shows micropillar deflection and tensile force to measure clot contractile force and stiffness, respectively (Chen et al., 2019).

Microfluidic viscometers have been shown to enable highly precise physiological measurement of blood coagulation with using just a few microliters of blood. For instance, they were applied in order to study the mechanisms underlying thrombus formation in micro channels resembling the human microvasculature (Colace et al., 2013; Branchford et al., 2015; Nagy et al., 2017; Zhang and Neelamegham, 2017). There are different types of microfluidic viscometers that work based on the measurement of pressure/velocity, surface tension, vibrating element frequency, and drag force (Gupta et al., 2016; Judith et al., 2018). Jain et al. developed a shear gradient-activated microfluidic device that mimics a network of stenosed arteriolar vessels for measuring whole blood coagulation and platelet function. Figure 2A shows the microfluidic system consisting of a pressure sensor and a syringe pump to push and pull the blood inside the microfluidic channel (Jain et al., 2016a). Kim et al. demonstrated a smart phone based viscometer that monitors the interfacial width in images from a Y shaped channel, while the ratio of the pressure in the blood channel (sample) over the pressure in PBS channel (reference) is correlated with the width ratio of the sample flow W/W_total_ (Kim et al., 2018). Li et al. developed a paper based microfluidic lateral flow device (see Figure 2B) in which the traveling distance of RBC cells in a cellulose membrane was correlated with the blood clotting time (Li et al., 2014). Yeom et al. studied blood viscosity and platelet adhesion at the same time based on an H shaped microfluidic channel while the pressure ratio was correlated to the change of interfacial width between phosphate buffered saline and blood sample (Yeom et al., 2016). Chen et al. demonstrated a microfluidic device to measure micro clot elasticity based on the micropillar arrays under fluid shear. Figure 2C shows the device which consists of a top microfluidic channel layer, a middle PDMS substrate with the micro-tissue array, and a bottom stretchable membrane (Chen et al., 2019). This device has facilitated for the first time the study of clot mechanics under biochemical treatments and shear flow, whose results suggest that the two stimulants strongly affect clot remodeling and stiffness independently.

Propagated ultrasound velocity affected by the coagulation process inside the clotting blood is the main measurement parameter in this technology. Rachel et al. studied acoustic properties of the clotting blood with a high frequency ultrasound transducer; red blood cell aggregation was extracted from backscattering parameters and liquid gel transition phase from the sound velocity (Libgot-Callé et al., 2008). Machado et al. measured human clotting time of blood plasma by using scattered ultrasound from spherical particles kept in motion (Machado et al., 1991). Doppler ultrasound was applied to measure power and flow velocity which were found to be increasing and decreasing, respectively, during the blood coagulation (Huang et al., 2005b). Ultrasound has been used also for real time monitoring of coagulation after injecting fibrinolytic drugs (Ivlev et al., 2019).

### POC Tests Based on Photoacoustic Measurements

Photoacoustic imaging is based on the laser-induced acoustic vibrations in tissue which inherits both the advantages of deep penetration of acoustic waves and high contrast of optical imaging (Wang and Hu, 2012). *In vivo* photoacoustic (PA) based detection has been used for real time monitoring of circulating clots, blood coagulation monitoring in tissue (Larina et al., 2005; Larin et al., 2005), and therapeutic heparin monitoring (Galanzha et al., 2011; Juratli et al., 2013; Wang et al., 2016; Jeevarathinam et al., 2019). Ananthakrishnan et al. developed a cellulose-based photoacoustic sensor with Nile blue A loaded onto polyethylene glycol (PEG) modified Whatman filter paper substrates. Fluorescent molecules such as Nile blue A and methylene blue can be used as a dye in photoacoustic detection based on dual wavelengths pump-probe excitation, which in turn modulates the amount of thermalized energy, and hence the PA signal amplitude (Märk et al., 2015). In the cellulose-based heparin sensors, heparin via the formation of heparin-Nile blue A aggregation leads to higher PA signal because of electrostatic interaction. This higher photoacoustic activity is due to decreased fluorescence, reduced degrees of freedom, and poor heat transfer to the solvent. This sensors showed a detection limit of 0.28 IU/ml heparin in human plasma and 0.29 IU/ml in whole blood with a linear response (Pearson's *r* = 0.99) from 0 to 2 IU/ml heparin in plasma and blood samples were reported.

Combined ultrasound and photoacoustic techniques have been employed for *in vivo* detection of stage deep vein thrombosis (Karpiouk et al., 2008). Circulating blood clots (CBCs) or emboli as a result of thromboembolism can block vessels in the body, and they can be caused by surgery, cancer, radiation, and infection. In this regard, photoacoustic technique is very promising because it can be used both in real time and *in vivo*.

### POC Tests Based on Electrical and Electrochemical Measurements

The electrical impedance of the blood is related to plasma resistance, cell membrane's resistance, and capacitance (Zhao, 1993). In normal blood, the impedance of plasma is dominant while during the coagulation process, aggregation of RBCs leads to an impedance network (increase of the resistance) of the cells, which are connected to each other via fibrin fibers, and it causes a drastic increase in the whole blood impedance. In addition, this complex impedance increases with the frequency as the induced interconnected resistance/capacitance between cells in the formed network plays the main role in blood resistance. However, the resistance of plasma and red blood cells decreases with increasing frequency (Pradhan et al., 2012). There have been many studies to measure blood coagulation with impedance measurements. Ur et al. showed that the changes in the blood impedance due to coagulation is reproducible and can be used for blood clot monitoring (Ur, 1970). Advances in the integrated electronic circuits along with the emerging of the microfluidic platforms have enabled the usage of this technique to be more practical and cost effective for blood coagulation monitoring (Berney and O'Riordan, 2008; Lei et al., 2013; Ramaswamy et al., 2013). Another type of the electrical measurement based on carbon nanotube strain sensors has been reported as well. These strain sensors are embedded within the micropillars which can sense mechanical force due to clotting blood droplet and the resulting force is transferred to the resistive nanotube strain sensor (Li et al., 2018).

As thrombin (which converts soluble fibrinogen to fibrin) activation leads to the blood clot, electrochemical sensing of this enzyme is also widely used for the thrombin clotting time measurement (Hianik et al., 2005). Yang et al. reported a molecular imprinted polymer (MIP)-aptamer based sensor for voltaic measurement of thrombin with linear range of response from 2.5 × 10^−9^ to 1.3 × 10^−6^ mg/mL (Yang et al., 2019). Xiao et al. demonstrated a novel, selective, and rapid aptamer-based (E-AB) sensor (consists of a modified electrode with surface immobilized, redox-tagged DNA aptamers) for detection of thrombin in blood serum (Xiao et al., 2005). Suprun et al. developed AuNP redox-based platform for protein detection when thrombin-thrombin interaction causes a cathodic peak in cyclic voltammetry output (Suprun et al., 2008). Aptamer–gold nanoparticles–horseradish peroxidase (aptamer–AuNPs–HRP) hybrid was shown to amplify the detected thrombin signal by taking the advantage of AuNPs, and oxidation of HR catalyzed by HRP (Zhao et al., 2011). Cheng et al. developed a dual-readout colorimetric/electrochemical aptamer-based thrombin sensor consisting of platinum nanoparticle modified metal-organic framework (type Fe-MIL-88) providing a low detection limit (0.33 fM) for the electrochemical method and 0.17 pM for the colorimetric method (Cheng et al., 2019).

## Nanomaterials for Blood Coagulation Monitoring and Treatments

Growing field of nanomedicine in recent decades has created a need for the investigation of nanomaterials' biocompatibility including their anticoagulation properties. Anticoagulation properties of these nano materials such as metal nano particles (gold, silver, and platinum; Ehmann et al., 2015; Dakshayani et al., 2019), metal oxide nano particles of titanium oxide (Huang et al., 2003) and zinc oxide (Huang Z. et al., 2011), carbon nano materials of graphene oxide and carbon nanowires (Wang et al., 2012; Haung et al., 2013), and modified polymeric nano materials have been studied and they were shown to be used successfully in the treatment and detection of hemostasis diseases. Nano particles can specifically interact with the coagulation system or avoid to interact. Generally, this interaction can be in two different ways: contact with plasma coagulation factors or interaction with cells, such as epithelial cells, monocytes, and platelets (Matus et al., 2018).

**Figure 3:**
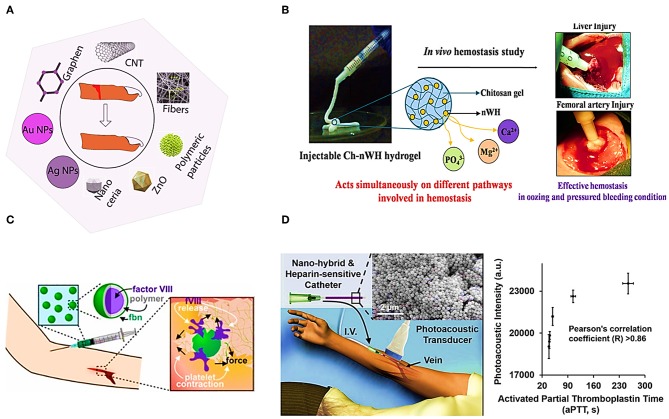
Illustrates different applications of nanomaterials. **(A)** Application of nanomaterials in wound healing. **(B)** Targeted drug delivery system utilizing polyelectrolyte multilayer capsule (Muthiah Pillai et al., 2019). **(C)** Platelet-hybridized system for targeted delivery of hemostatic agents utilizing polyelectrolyte multilayer capsules (Hansen et al., 2017). **(D)** APTT test based on Silica nanoparticles and photoacoustic detection technology (Wang et al., 2016).

Herein we review the usage of some nanomaterials for blood coagulation detection and treatment. Figure 3A shows the usage of different nanomaterials for wound healing. Polymeric nanoparticles, metallic nanoparticles, zinc oxide Nps, Nanocreia, and electrospun nanofibers have been used to stop bleeding and wound healing (Kalashnikova et al., 2015). For instance, electrospun N-Alkylated chitosan (NACS) fibers have been studied as an effective hemostasis agent. NACS can be used to stop bleeding by converting whole liquid blood into a gel immediately It has been explained that, the main mechanism of clotting by Ch-based hemostatic agent acts is by adhering and physically sealing the bleeding wound based on mainly the electrostatic interaction between positive charge of protonated amine group of Ch and negative charge of erythrocytes cell membranes. This fibers have been shown to be in favor of the activation of coagulation factors and platelets (Wang et al., 2018). Pillai et al. reported Chitosan hydrogel incorporated with nano Whitlockite [WH: Ca_18_Mg_2_ (HPO_4_)_2_(PO_4_)_12_] including approximately up to 20 wt% of the inorganic phase of human bone. WH consists of Ca^2+^, Mg^2+^, and ${\text{PO}}_{4}^{-3}$ ions which activate different coagulation factors involved in coagulation cascade. Figure 3B illustrates the mechanism of inducing hemostasis by Ch-nWH composite hydrogel, and its rapid and effective hemostasis in *in vivo* experiment (Muthiah Pillai et al., 2019). Figure 3D shows a hybrid of agar gel and thiol-coated silica nanoparticles immobilized with methylene blue, and the result of aPTT test to monitor heparin. Methylene blue is a Federal Drug Administration (FDA) approved contrast dye agent responding to heparin. This hybrid has a significant and dose-dependent increase in photoacoustic signal in the presence of both heparin and low molecular weight heparin (LMWH) (Wang et al., 2016). Responsive thrombus-specific theranostic (T-FBM) nanoparticles that could provide H_2_O_2_-triggered photoacoustic signal amplification have been shown to be used as an antithrombotic nanomedicine (Jung et al., 2018).

Sensing of thrombin activity assisted by nanomaterials is the another main application of them in coagulation monitoring (Lin et al., 2013). They have been used as synthetic biomarkers in Urine for non-invasive monitoring of intravascular thrombosis and cancer (Lin et al., 2013; Warren et al., 2014). To detect thrombin, thrombin-sensitive peptide substrates are conjugated with nanoparticles. For instance, thrombin-activated near-infrared fluorescence (NIRF) probe consists of a thrombin-cleavable peptide spacer and contained a terminal fluorescence reporter which is quenched when conjugated to a biocompatible delivery vehicle. Following thrombin addition, the probe is activated within minutes. The changes in the relative thrombin activity is measured through fluorescent intensity (Tung et al., 2002). In addition, different types of nanomaterials such as magnetic (Khurshid et al., 2017), metallic, and metal oxide nanomaterials have been employed for blood clot detection as explained in the electrochemical sensing.

Furthermore, there are many studies on treatment and imaging of acute thrombi by nanoparticles and nanotheranostics. In fact conjugation of the nanoparticles with chemical agents interacting with the blood coagulation elements enables treatment and imaging. Heparin conjugated nano particles is one example. Xiang et al. employed ROS-responsive heparin-DOCA/PVAX nanoparticles to inhibit thrombus formation (Xiang et al., 2019). In their work, by using nanoparticles that contain anti-inflammatory polymer (copolyoxalate containing vanillyl alcohol, PVAX) and anti-thrombotic heparin derivative deoxycholic acid (Hep-DOCA), they manipulated the inflammation-associated coagulation. Voros et al. reported TPA immobilization on iron oxide nanocubes to accelerate clot lysis (Voros et al., 2015). Xin Yu et al. used a novel fibrin-targeted paramagnetic nanoparticle contrast agent for high-resolution MRI characterization of human thrombus (Yu et al., 2000). McCarthy et al. developed magneto-fluorescent crosslinked dextran-coated iron oxide nanoparticle platform as a multifunctional nano agent for thrombus-targeted fibrinolytic therapy (McCarthy et al., 2012).

Moreover, there are some of the nano hemostasis materials commercially available or in the phase of FDA approval such as silver NPs (various FDA-approved silver-based formulations have been used for chronic wounds). For instance, Acticoat™ produces wound dressing consists of silver nanoparticle wich can be released to the wound. Electrospun nanofibers (Rolandi et al., 2014), synthetic fibrin polymers (Chan et al., 2015), and nanocarriers for drug delivery (Nethi et al., 2019) are other nanostructured materials which are in FDA approval phase. In addition, thrombin@Fe_3_O_4_ nanoparticles which has been used as a hemostatic agent in internal bleeding (Shabanova et al., 2018), PolySTAT, chitosan nanoparticles, and electrospun fibers are hemostasis materials approved by FDA. One commercial device for wound healing is SpinCare which is a portable electrospinning device and can produce nanofibers containing drugs such as antibiotics, as well as antibacterial compounds, collagen, silicon, and other substances that may facilitates healing a wound.

## Commercial POCT Devices for Blood Coagulation Monitoring

**Figure 4:**
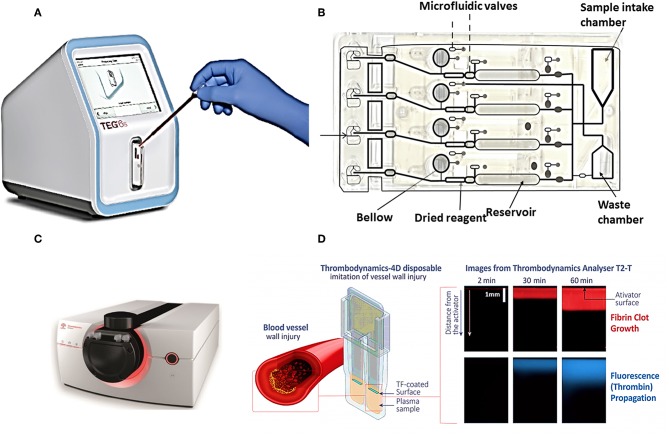
Two commercial devices for viscoelastic (TEG-6S system) and thrombodynamic analysis (Thrombodynamics Analyzer System). **(A)** Image of TEG® 6s system TEG-6S analyzer and **(B)** microfluidic TEG6s cartridge (images courtesy of Haemonetics Corporation, USA). **(C)** Thrombodynamics® Analyzer, schematic of a plastic cuvette, and an inserted activator for Thrombodynamic measurement, **(D)** the images from growth of a clot initiated with the activator, and spontaneous clots in the bulk of the sample (images courtesy of Hemacore Corporation, Russia).

Commercial POCT devices for blood coagulation monitoring can be divided in two categories: (1) devices for standard diagnostic blood clotting tests such as PT/INR, and APTT tests, (2) devices which measure directly the viscoelastic properties of clotting blood. Table 2 lists some commercial coagulation analyzers. Hand-held devices to test blood clotting tests include Xprecia Stride™ Coagulation System (Piacenza et al., 2017) and **CoaguChek** (Plesch et al., 2008) which are working based on electrochemical sensing, Optical Coagulation Analyzer (OCG-102) which is working based on optical technology, and COAGMAX is a MEMS device. This portable devices allow monitoring of clotting status in homes, and also facilitate the rapid assessment of clotting status in all clinical scenarios including operating and emergency rooms. On the other hand, the second category of devices provide more information and coagulation parameters similar to TEG/Rotem devices. For instance, TEG-6S is a miniaturized version of TEG system which consists of a microfluidic cartridge and piezoelectric resonators (Gurbel et al., 2016), Thrombodynamics® Analyzer provides thrombodynamics analysis (TD) based on the analysis of thrombin distribution with optical and fluorescent imaging (Sinauridze et al., 2018), and Quantra™ Hemostasis Analyzer is a new developed device for evaluation of hemostasis providing clotting time, clot stiffness, platelet, and fibrinogen contributions to clot stiffness based on the ultrasonic technology (Ferrante et al., 2016; Baryshnikova et al., 2019). Figures 4A,B shows the commercial devices TEG-6S and Thrombodynamics® Analyzer respectively.

**Table 2:** Commercial available POCT devices.

**Device/company**	**Technology**	**Blood sample**	**Sample volume**	**Coefficients of variation (CV)**	**Tests**
Hemochron Signature/Accriva Diagnostics, Inc part of instrumentation laboratory (ITC)	Optical-Channel microfluidic platform	Citrated whole blood or a finger stick sample	≥10 μL	≤ 10%	PT, aPTT, ACT, ACT-LR, INR (0.8 to 10.0)
Xprecia Stride™ Coagulation System/Siemens Medical Solutions USA, Inc.	Electrochemical sensing with amperometric detection of thrombin activity	Whole blood	6 μL	<5%	PT/INR
CoaguChek/Roche Diagnostics	Electrochemical sensing	Fingerstick, Whole blood	≥8 μL	<5%	INR
Quantra™ Hemostasis Analyzer/HemoSonics	Ultrasound	Whole blood	(<1 ml)	<5%	Clotting time, clot stiffness, and platelet and fibrinogen contributions to clot stiffness.
Optical Coagulation Analyzer (OCG-102)/Wondfo	Optical	Whole blood	20 μL	<5%	PTINR/APTT/ACT/TT/FIB
Thrombodynamics® Analyzer/Hemacore	Optical and fluorescent imaging	Plasma	25 μL	<10%	PT/APTT/INR Thrombodynamics (TD) assay
TEG-6S/Haemonetics	Microfluidics-MEMS	Whole blood	360 μL	<2.1%	Clot viscoelasticity
Coagmax/Microvisk	MEMS	Fingerstick	5 μL	= 10.1%	PT/INR

The TEG-6S is a fully automated diagnostic platform (see Figures 4A,B), a four-channel microfluidics cartridge is used for parallel analysis of 4 samples. The cartridge is loaded with (~0.4 ml volume) with a pipette. Reagents are dried and resident within each channel, and after moving blood by the microfluidic valve and bellow action they are mixed. After that, approximately 20 μl of mixed sample is transferred to the terminus of each microchannel, where clotting is monitored. Waste chamber is for the excess of the sample. Thrombodynamics® Analyzer provides thrombodynamics (TD) analysis (see Figures 4C,D), a new global coagulation test which measures the actual anticoagulant activity of the drug in a particular patient rather than concentration of heparin in the blood. In this system uses time-lapse video-microscopy to extract and quantify spatiotemporal parameters of fibrin clot growth/lysis. The clot growth curve is obtained from both optical and fluorescent imaging. As the Figure 4D shows, coagulation is initiated by an activator fixed in space and extends in a thin layer of non-stirred plasma. Clot size is measured through Light scattering while it increases in an area against time. Therefore, this system model imitate *in vitro* physiological and pathophysiological processes closer to *in vivo* conditions than other homogeneous global tests. The parameters extracted from clot growth curve are: Tlag (the time to the start of clot growth); Vi (initial) and V (stationary) spatial clot growth rates (the slopes of the clot size curve vs. time for the segments of 2–6 min and 15–25 min from the clot growth start for Vi and V, respectively); and CS (the clot size at 30 min after coagulation activation). Two other important parameters may also be measured: the maximum optical density of the formed clot (D), which characterizes the clot quality, and the time of appearance of spontaneous clots in the sample (Tsp) (Sinauridze et al., 2018). Competitive advantages of thrombodynamics test include: (1) it enables to evaluate the efficacy of anticoagulant drugs including newest version of direct oral anticoagulants (DOACs), (2) enables to evaluate efficacy of anti-platelet therapy, (3) enables to monitor and study thrombin generation.

Although recent developments in the development of blood coagulation analyzers are promising, still there are a high potential for developing novel monitoring and therapeutic technology in the future based on photoacoustic detection and nanotechnology. The aforementioned research papers showing the powerful methods of fluorescent imaging and photoacoustic detection is capable of *in vivo* measurements while there are some FDA approved dyes that can be used for heparin detection. On the other hand, microfluidic platforms which some are reported already in research papers have been patented and they may be produced in the future. Therefore, this review also help industry sectors for possible future products and trends which at the same time save the time and money for new start-up for developing new platforms based on this review.

## Future Perspectives

### Novel Anticoagulant Therapies

**Figure 5:**
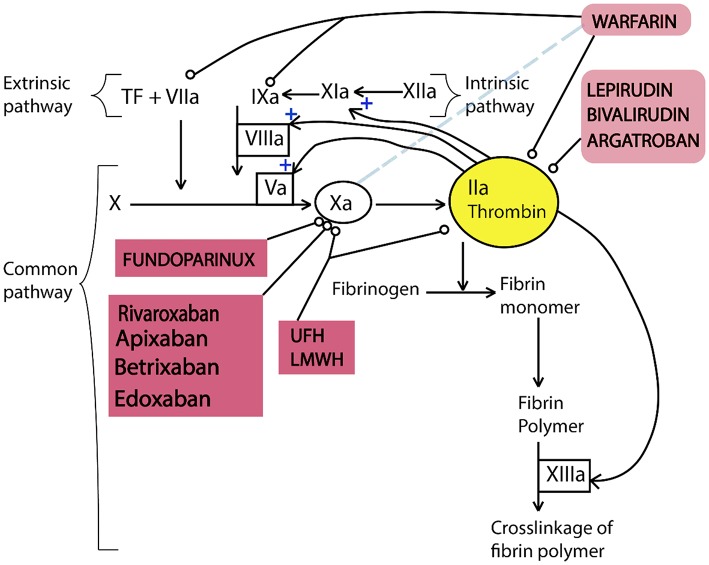
Simplified diagram illustrating the function of anticoagulants in the coagulation cascade. Coagulation cascade includes three pathways of intrinsic, extrinsic, and common pathways. In this diagram, thrombin has positive effect on the further activation of the intrinsic and common pathways. All anticoagulants affect factors of the intrinsic and common pathways except warfarin which reduces the rate of hepatic synthesis of factor VII. This figure is reproduced from American Journal of Neuroradiology, 2012 (Hussein et al., 2012).

Anticoagulants are used to treat blood clots during thromboembolic events which do not have specific signs and consistent symptoms (Flato et al., 2011). For example, this agents are used to prevent and treat venous thromboembolism, for stroke prevention in atrial fibrillation, embolism prevention in heart failure, and in the management of atrial fibrillation. These new oral anticoagulants are in various phases of clinical development. In the Figure 5, the targets of anticoagulants in the coagulation cascade has been illustrated. Direct thrombin inhibitors (DTI) prevent thrombin activity in free plasma and at the thrombus. Lepirudin, Bivalirudin, argatroban, and Fondaparinux are in this category of anticoagulants (Hussein et al., 2012). Consequently, they block conversion of fibrinogen into fibrin, decreasing thrombin generation which affects the amplification and propagation of coagulation. On the other hand, Direct factor Xa inhibitors (DFXaI) directly affects Factor Xa without effects on other intrinsic/extrinsic coagulation pathways. Rivaroxaban, apixaban, edoxaban, and betrixaban are in this category of anticoagulants (Flato et al., 2011).

With the introduction of novel anticoagulant therapies, the validity and applicability of current coagulation measurement techniques have been in question (Funk, 2012). The new generation of anticoagulant therapies involving dabigatran, rivaroxaban, apixaban, edoxaban, and betrixaban were designed to minimize the requirement for monitoring or dose adjustment of the patient (Mousa, 2010). However, while with these novel anticoagulants, the need for monitoring might be reduced, it will never go away for patients with special needs such as kids, pregnant woman and senior people (Dale et al., 2016). New devices have been shown to be capable of measuring and studying effect of both types of DOACs: direct thrombin inhibitors and Anti -Xa(s), although this devices have not been employed in all clinical centers. Therefore, the applicability of already established methods needs to be verified with these novel drugs and new methods and parameters which will enable their monitoring will need to be established.

### Multiplexed Sensing

Electrochemical biosensors embedded inside microfluidic chips facilitate multiplexed sensing of different parameters such as pH, oxygen, glucose, lactate, and chloride (Ehgartner et al., 2016). In addition, microfluidic centrifugal technology has enabled miniaturization of typical laboratory processes such as blood plasma separation (Haeberle et al., 2006) and enzyme-linked immunosorbent assay (Lai et al., 2004). In this regards, combining these novel platforms with microfluidic viscometers lead to the development of multiplexed microfluidic chips for blood coagulation monitoring and other blood tests. Moreover, different fluorescent probes provide monitoring of different blood coagulation factors such as thrombin (Tung et al., 2002) and fibrin (Hara et al., 2012). Multiplexed sensing for both blood coagulation analysis and other biochemical parameters is promising for developing low cost and multiplexed blood assessments.

### Artificial Intelligence in Diagnosis and Monitoring

With the advancements in artificial intelligence (AI) and machine learning, algorithms that can track multiple parameters simultaneously throughout a treatment/diagnosis and find out patient specific patterns that can aid in pinpointing proper treatment or underlying causes will become one of the biggest developments in coagulation measurement technologies in the upcoming years (Chapman et al., 2015; Hansen et al., 2016; Krumm et al., 2018). Especially with the challenges set by the novel anticoagulant technologies for the current measurement techniques and the validity and applicability of diagnostic parameters such as INR, simultaneous observation of multiple parameters or complicated patterns within them may be necessary for proper observation of patients with special needs. For these special cases, AI and machine learning based algorithms may very likely find place within future novel POC blood coagulation measurement technologies in the upcoming years (Ngufor et al., 2015; Krumm et al., 2018).

## Conclusion

Blood coagulation monitoring with high level of accuracy and reliability for anticoagulant drug dose adjustments (e.g., for heparin and warfarin), studying effects of the drugs, and checking the risks in surgeries for the patients is highly demanded. Viscoelastic assessments, optical (scattering and fluorescent imaging), and electrical impedance measurement are frequently used for both evaluating pharmacological treatments and diagnosing the blood coagulation abnormalities. Recent advances in microfluidic technology has enabled the researchers to simulate the blood coagulation process in physiological conditions and study the events in the molecular level. Moreover, the fluorescent imaging and targeting different particles with fluorescent probes in microfluidic channels facilitate understanding of the interactions and origin of defects with a remote, accurate, and multiplexed manner. Other platforms like centrifugal microfluidic devices can be used for future multiplexed analysis of blood as it facilitates the separation of different blood components. Moreover, we discussed different nanostructured materials for the detection and treatment blood coagulation purposes which helps developing future monitoring and control of hemostasis at the same time in *in vivo* condition. In this paper, we covered different technologies for coagulation sensing and their working principle, some recent commercial devices, and possibilities of continuous *in vivo* hemostasis monitoring.

## Author Contributions

MM, AE, and OY wrote the article, read, and approved the final version of the manuscript.

### Conflict of Interest

The authors declare that the research was conducted in the absence of any commercial or financial relationships that could be construed as a potential conflict of interest.
